# Research on RARs in neurodegenerative diseases: A bibliometric analysis

**DOI:** 10.1097/MD.0000000000049472

**Published:** 2026-06-26

**Authors:** Ting Wu, Tengyu Zhang, Ahmad Khaled Harb, Xiaojie Zhai, Wei Cui, Yunfei Cao, Xiang Wu

**Affiliations:** a The First Affiliated Hospital of Ningbo University, Ningbo, China; b Translational Medicine Center of Pain, Emotion and Cognition, Zhejiang Provincial Key Laboratory of Pathophysiology, Health Science Center, Ningbo University, Ningbo, China; c Beilun District People’s Hospital of Ningbo, Ningbo, China.

**Keywords:** bibliometric analysis, data visualization, neurodegenerative diseases, retinoic acid receptors

## Abstract

**Background:**

In recent decades, thousands of research articles on neurodegenerative diseases (NDs) have been published. Retinoic acid and its analogues play crucial roles in biological processes such as cell proliferation, differentiation, and apoptosis through their interaction with retinoic acid receptors (RARs). While the involvement of RARs in NDs has attracted increasing interest, a further understanding of the current state and future trajectories of RARs research within this field needs to be explored. This study aims to provide a systematic overview through bibliometric and visual analysis.

**Methods:**

Original research and review articles concerning RARs in NDs were systematically retrieved from 3 databases: Web of Science Core Collection, Scopus, and PubMed. Subsequent statistical analysis and graphical representation of data on country, institution, authorship, journal, and key terms were conducted using advanced software like VOSviewer, CiteSpace, and the bibliometric toolbox within the R programming language.

**Results:**

A total of 1094 articles were included in the analysis, with the United States leading in both publication output (n = 254) and total citations (TC = 17,102), followed by China and Germany. The United States also demonstrated the highest total link strength (90), indicating its central role in international collaborations. The University of California System was the most prolific institution. Keyword analysis revealed core research themes including “retinoic acid,” “neurodegeneration,” “neuroinflammation,” “oxidative stress,” and “neuronal differentiation,” with recent shifts toward mechanisms involving microglia, the blood–brain barrier, and translational models.

**Conclusion:**

Research on RARs in NDs represents a dynamically growing and interdisciplinary field. The USA has contributed most substantially to the literature, underscoring the importance of international and institutional collaboration. Current and emerging research hotspots focus on intracellular calcium, cancer, tau protein, and inflammation, highlighting pathways with therapeutic potential. Future studies should further elucidate molecular mechanisms, integrate advanced technologies such as single-cell sequencing, and accelerate the translation of RAR-related findings into clinical applications.

## 1. Introduction

Neurodegenerative diseases (NDs) represent a diverse group of complex disorders characterized by progressive neuronal loss and degeneration across multiple regions of the nervous system.^[1]^ One potential therapeutic avenue involves targeting programmed cell death pathways.^[2]^ Uncontrolled neuroinflammation, particularly driven by microglial activation, is considered a key driver of chronic disease progression, often establishing a self-sustaining cycle that may be a promising target for disease-modifying interventions.^[3]^

The global aging population is expected to increase the prevalence of NDs, placing a heavy burden on patients’ families and socioeconomic status.^[4]^ To address this growing challenge, researchers are actively searching for new therapeutic targets and strategies. Among these, retinoic acid receptors (RARs) have attracted significant interest due to their important roles in nervous system development and function. RARs belong to the steroid and thyroid hormone receptor superfamily and play a key role in cell development, differentiation, and maintaining homeostasis.^[5,6]^ This receptor family includes 3 subtypes: RARα, RARβ, and RARγ, which form heterodimers with retinoid X receptors.^[7,8]^ Retinoic acid, synthesized in specific locations, regulates transcription by binding to nuclear RARs and acting on retinoic acid (RA) response elements near target genes.^[9]^ Research shows that activating RARα can alleviate neuroinflammation after subarachnoid hemorrhage by promoting the polarization of microglia from the M1 to the M2 phenotype and modulating the Mafb/Msr1/PI3K-Akt/NF-κB signaling pathway.^[10]^ RARβ can inhibit glial scar formation and promote spinal cord regeneration by directly regulating phosphatase and tensin homolog activity in neurons and influencing astrocytes via exosomes.^[11]^ Additionally, RARγ specifically mediates the teratogenic signals of RA and is closely linked to neural plate patterning and neural tube formation.^[12]^ Furthermore, emerging experimental studies underscore the therapeutic potential of RAR agonists in neurodegenerative models. For instance, RARα agonists such as all-trans retinoic acid have been shown to upregulate the α-secretase a disintegrin and metalloproteinase 10 (ADAM10), promoting the nonamyloidogenic processing of amyloid precursor protein and reducing β-amyloid accumulation in models of Alzheimer disease.^[13]^ In Huntington disease models, activation of RARβ exerts neuroprotective effects on striatopallidal medium spiny neurons, potentially by preserving mitochondrial function and mitigating neurodegeneration.^[14]^ RARα agonist AM80 efficiently combats age-induced memory decline by activating the ADAM10–Notch-Hes5 neurogenerative pathway in the hippocampus.^[14]^ Activation of RARα prevents surgery-induced cognitive impairment by inhibiting neuroinflammation; this occurs via the inhibition of the TLR4/Myd88/NF-κB pathway and restoring synaptic proteins by stimulating the brain-derived neurotrophic factor (BDNF)/ERK/CREB pathway.^[15]^

Over the past several decades, a great deal of research has been dedicated to the investigation of NDs and their relationship with RARs. However, studies on research trends within related fields remain relatively unexplored. Bibliometrics is a widely recognized methodology that helps to analyze development and research patterns in specific fields.^[16]^ This comprehensive resource offers researchers an all-encompassing perspective on fundamental data and evolving patterns, facilitating their evaluation of both the quantity and quality of scholarly works concerning existing topics, institutions, and geographical areas.^[17]^ To achieve this goal, we methodically categorized and assessed data based on country, authorship, journal, and affiliations. Furthermore, we conducted an in-depth analysis of keyword prevalence and temporal patterns, visualizing them in bibliometric maps to forecast future research trends.

## 2. Materials and methods

### 2.1. Literature sources and search strategy

To obtain comprehensive and reliable literature data, this study simultaneously utilized 3 databases – Web of Science Core Collection (WOSCC), Scopus, and PubMed – as primary search sources. WOSCC is widely used in medical and life sciences research due to its high-quality, structured citation system. This study exclusively incorporates the Science Citation Index Expanded and Emerging Sources Citation Index to ensure the academic representativeness and consistency of the included literature. As one of the world’s most comprehensive peer-reviewed literature databases, Scopus provides interdisciplinary and systematic literature support, thereby expanding the scope of this study’s search. PubMed, developed by the U.S. National Library of Medicine, is the most central literature database in the biomedical field and a vital source for highly relevant medical literature. Therefore, conducting a systematic search across these 3 databases helps comprehensively present the full landscape of research developments in NDs and RARs.

Based on the selected databases, this study constructed independent yet structurally consistent retrieval strategies across the 3 databases. The retrieval themes centered on 2 core concepts – “retinoic acid receptors” and “neurodegenerative diseases” – with term combinations built using wildcards and Boolean logic to maximize coverage of relevant term variants. To enhance topic relevance, the search scope was uniformly restricted to the title, abstract, and author keywords fields. Additionally, automatically generated index terms (e.g., WOSCC’s Keywords Plus) were excluded to prevent bias introduced by nonmanually annotated terms. The WOSCC search was limited to the TS field; Scopus covered the TITLE-ABS-KEY fields; PubMed was restricted to the Title/Abstract fields. All searches were restricted to English-language literature and included only Article and Review types. The final search date was January 21, 2026. Complete search terms are detailed in Supplementary File 1, Supplemental Digital Content 1.

**Figure 1.**
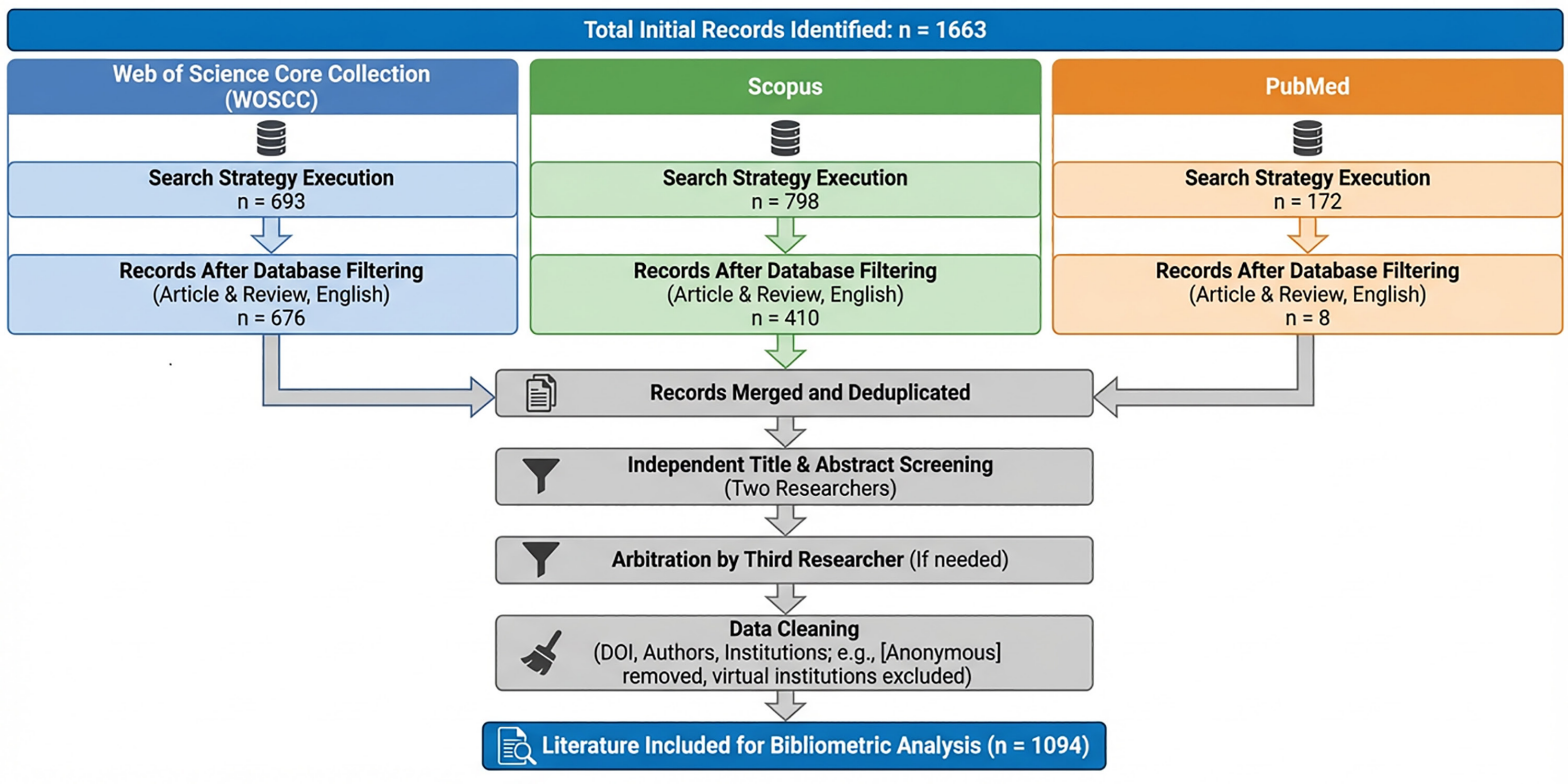
Flowchart for searching, sorting, and choosing articles to include in the literature.

This search yielded 1663 initial documents (WOSCC: 693; Scopus: 798; PubMed: 172). Two researchers (A and B) then independently screened all documents for relevance. Any disagreements during screening were resolved by a 3rd researcher (C), who made the final decision. After excluding irrelevant studies, the literature data were downloaded in plain text and comma-separated values formats, including comprehensive information such as titles, authors, institutions, countries, publication years, abstracts, keywords, and references. To standardize data structure, Python (version 3.11) was used to convert Scopus comma-separated values files into plain text formats consistent with WOSCC and PubMed (full-record and reference formats). Data cleaning was also performed using Python (version 3.11), involving the following key steps: removing records with “[Anonymous]” in the author field; excluding virtual institutions such as “Egyptian Knowledge Bank (EKB)”; and merging duplicate institutional names. Prior to importing the cleaned dataset into VOSviewer (v1.6.20) and CiteSpace (v6.3.R1) for co-authorship, co-occurrence, and co-citation analyses, the author and country/region fields were further standardized within the software environments to ensure disambiguation and consistency. For author names, the analysis settings were configured to enable the automatic counting of full-name variants as the same author. Additionally, high-frequency author names with similar spelling were manually reviewed and merged to minimize identification bias arising from formatting inconsistencies (e.g., punctuation, abbreviations). For countries/regions, names were unified based on the correspondence address field (e.g., “England,” “Scotland,” and “Wales” were grouped under “United Kingdom”). Historical or alternative geopolitical entity names (e.g., “Czechoslovakia”) were reclassified according to their current successor state(s) (e.g., “Czech Republic” or “Slovakia”), and corresponding data mergers were performed within the software to ensure the accuracy of the geographical collaboration network analysis. Ultimately, 1094 documents (WOSCC: 676; Scopus: 410; PubMed: 8) were included for subsequent bibliometric analysis. The document screening process is detailed in Figure 1.

### 2.2. Data collection and statistics

The retrieved publications were systematically compiled and converted into plain text format, encompassing complete records and cited references. Key details – including authors, publication year, journal names, H‑index, institutions, and geographic origins – were extracted for analysis. Statistical examination was conducted using Microsoft Excel 2019, VOSviewer (version 1.6.20), CiteSpace (6.3.R1), and Scimago Graphica.

Excel facilitated initial data organization, while VOSviewer and CiteSpace – both Java‑based visualization tools – were employed for in‑depth bibliometric mapping. VOSviewer supported co‑authorship, co‑citation, and keyword co‑occurrence analyses, along with burst detection and timeline views of terms. CiteSpace was used to identify salient keywords and generate knowledge maps composed of nodes and links. Scimago Graphica complemented these by enabling interactive geographic network visualization. In these maps, node size reflects frequency, and connecting lines indicate collaboration, co‑occurrence, or citation strength.

## 3. Results

### 3.1. Number of publications and their trend

**Figure 2.**
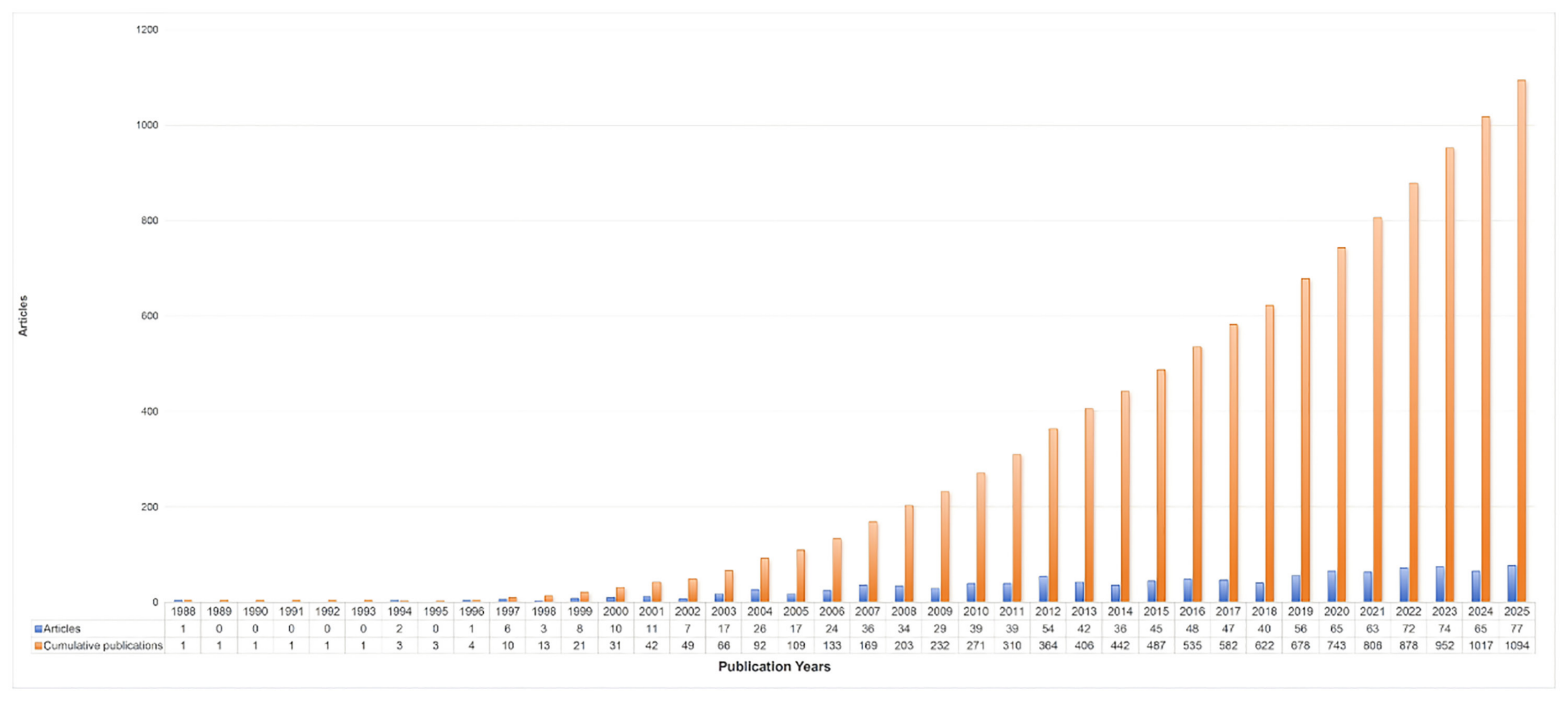
The number of yearly publications and patterns of increase.

Our search and filtering efforts yielded 1094 articles; as shown in Figure 2, we examined the number of publications (NPs) by year, with the earliest pertinent article being published in 1988. The publication count exhibits a cyclical upward trajectory, with fresh contributions being added annually.

### 3.2. Quantity and citations among different nations

**Figure 3.**
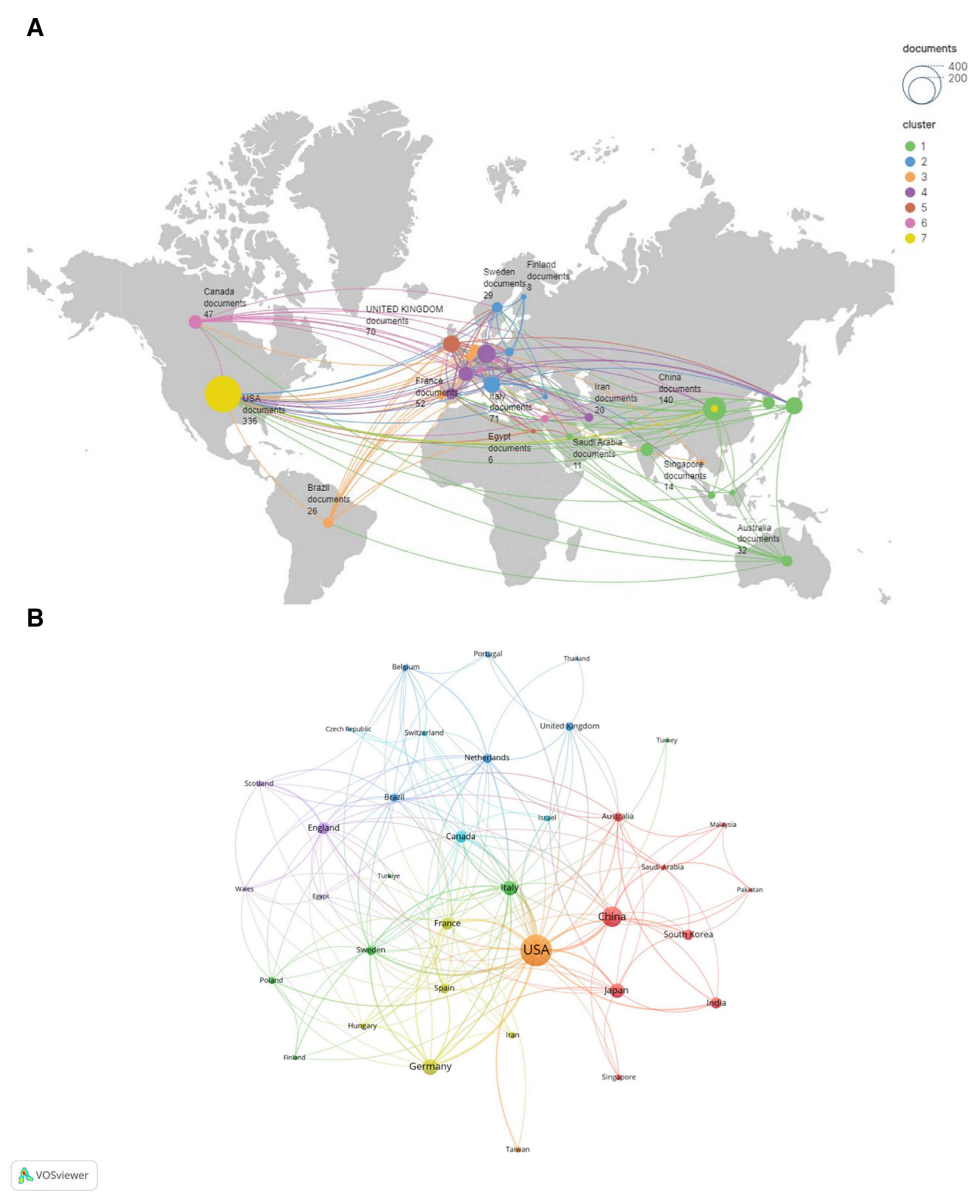
Quantity and citations worldwide. (A) Country/region paper map. (B) Networks of cooperation among nations worldwide.

A bibliometric analysis of the global research output in this field reveals a landscape characterized by a combination of high concentration and broad participation (Fig. 3A). The United States, with 336 publications, holds a dominant position, followed by China with 140 publications. Together, they form the leading echelon of knowledge production, accounting for over half of the total output, which underscores their substantial academic activity and resource investment in this research area. A second tier consists of several established research powers, including Italy (71), the United Kingdom (70), France (52), Canada (47), and Australia (32). These countries contribute at a similar scale, forming a stable core of supporting forces. The collective output from European nations (Italy, UK, and France) is particularly notable, reflecting the region’s strong research capacity. The remaining countries, such as Brazil (26 publications), Sweden (29), Iran (20), Singapore (14), and Saudi Arabia (11), among others, represent a broader network of participation. Their contributions, while smaller in volume, indicate widespread geographic interest in the research topic. It is noteworthy that the actual publication counts for some countries that appeared prominent in the original visualization – such as Finland (8) and Egypt (6) – are significantly lower than the visual impression might suggest. This highlights that in bibliometric analysis, precise quantitative data are more revealing of the true distribution than intuitive graphical representations. In summary, the research landscape in this field is primarily driven by the 2 core producers, the US and China, supported by a strong secondary group including multiple European nations, while maintaining a diffusely global scope of engagement. Knowledge production is highly concentrated in the core, despite widespread participation.

In terms of country collaboration, there is a clear variation in scientific output and international collaboration among countries (Fig. 3B, Supplementary Table 1, Supplemental Digital Content 2). The United States leads in the NPs (197), citation frequency (11,240), and total link strength (TLS) (90), reflecting high productivity, influence, and extensive international collaboration. China ranks second in publication volume (140), though its TLS (43) is relatively moderate, suggesting potential for increased collaboration. Several European countries, including Germany (TLS, 59), France (54), and Italy (51), demonstrate strong international linkage. Furthermore, some nations with limited publication numbers, such as Saudi Arabia (TLS, 17) and Hungary (17) – with 11 and 10 publications, respectively – still show notable participation in international cooperation, indicating their active engagement in specific research networks. Overall, the scale of scientific output does not always align closely with the intensity of international collaboration, highlighting differing roles and connectivity patterns within the global research landscape.

Next, we examined the frequency of publications across various nations, including both single-country and internationally coauthored publications (Table 1). Based on the affiliation country of the relevant authors, we compiled a list of the top 10 countries in terms of the number of citations for research works on the relationship between RARs and NDs. In terms of both citation count (total citations, TC = 17,102) and NPs (n = 254), the United States ranked first, followed by China (n = 140, TC = 5914) and Germany (n = 63, TC = 4184). Notably, France achieved a high average article citation rate (AAC = 77.60) despite having a relatively lower publication count (n = 33). In contrast, China, while ranking second in productivity, had the lowest (AAC = 42.20) among the top 10 countries. Japan (AAC = 37.30) and India (AAC = 23.30) also showed relatively lower average citation rates. There may be variations in the caliber of research publications published in various nations/regions, which could account for the discrepancies in publication count, total citation count, and average citation count per article.

**Table 1:** The top 10 productive countries.

Country	Publications	SCP	MCP	Freq	MCP Ratio	TC	Average article citations
USA	254	213	41	23.2	16.1	17,102	67.30
China	140	112	28	12.8	20	5914	42.20
Italy	65	44	12	5.9	21.4	2290	40.90
Germany	63	51	14	5.8	21.5	4184	64.40
UK	60	46	17	5.5	27	4226	67.10
Japan	56	51	9	5.1	15	2237	37.30
India	35	26	9	3.2	25.7	814	23.30
Korea	35	27	6	3.2	18.2	1246	37.80
France	33	24	8	3	25	2482	77.60
Canada	32	24	11	2.9	31.4	2003	57.20

Freq = number of all articles considering the collaboration with other countries; MCP = multiple country publications, SCP = single country publications, TC = total citation.

### 3.3. Collaboration among authors and analysis of referenced authors

**Figure 4.**
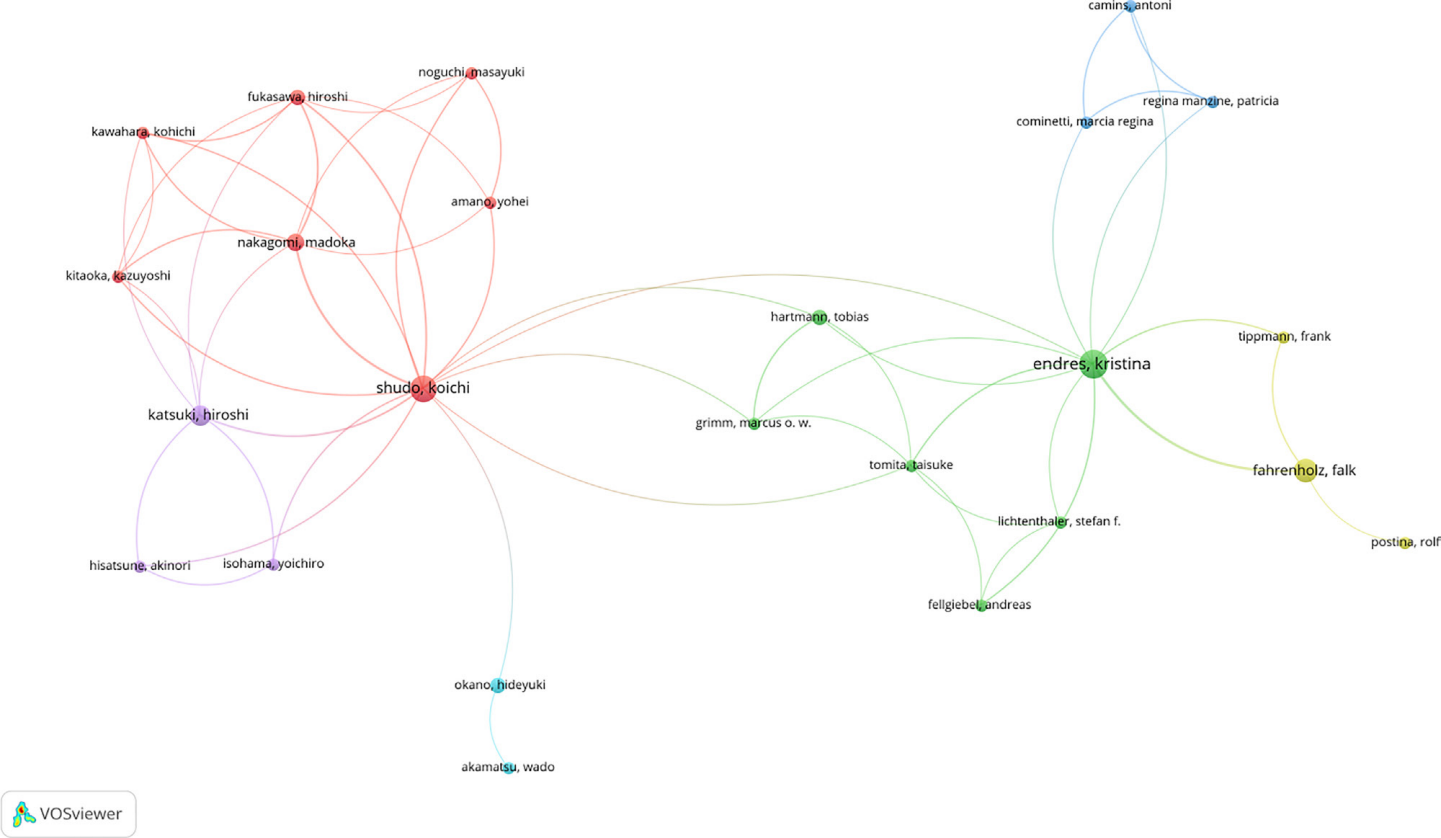
The writers who have authored at least 2 papers.

Based on the bibliometric analysis of co-authorship networks among authors with ≥2 publications in the field of RARs and NDs (Fig. 4), the network exhibits a modular structure with distinct yet interconnected research clusters, primarily focused on RA signaling, Alzheimer disease, and neuroinflammation. Key influential authors such as Shudo, Endres. demonstrate high centrality and citation impact, serving as hubs within and between clusters. The network reflects active international and interdisciplinary collaboration, particularly between European, Japanese, and North American research groups, facilitating the integration of basic retinoid biology with clinical neurodegeneration studies. Isolated high-impact authors and emerging collaborators suggest both established expertise and growing interdisciplinary engagement in this evolving research domain. Next, we provide an overview of the H-index and the total NPs authored by the top 10 authors (Table 2). With 4 publications published overall, ENDRES K and SHUDEN K exhibit the highest H-indices (11 and 10, respectively), coupled with substantial total citation counts (680 and 421), indicating their influential roles and sustained productivity in the domain. Authors such as Fahrenholz F and Zhang Y also show strong citation impact, though with moderate H-indices. Notably, while some researchers like Iverfeldt K and Krezel W started publishing earlier (publication year 1997), their m-index values are relatively low, suggesting a lower annual normalized impact. In contrast, more recent contributors such as Yang Y (publication year 2017) demonstrate a high m-index (0.6), reflecting a strong early-career influence per year. The collaboration network likely highlights interdisciplinary links between molecular neurobiology, receptor studies, and disease mechanism research, with core authors serving as hubs connecting smaller research groups. Such patterns emphasize the importance of collaborative efforts in advancing the understanding of RA signaling in neurodegenerative contexts. The presence of both long-standing and recently active authors suggests a dynamically evolving field with continuous knowledge integration and network expansion.

**Table 2:** The H-index and total number of articles contributed by the top 10 writers.

Author	Index			TC	NP	PY_start
	h	g	m			
Endres K	11	11	0.5	680	11	2005
Shudo K	10	11	0.435	421	11	2004
Fahrenholz F	8	8	0.364	669	8	2005
Wang Y	7	10	0.467	170	10	2012
Chen J	7	9	0.438	195	9	2011
Iverfeldt K	6	6	0.2	261	6	1997
Yang Y	6	6	0.6	107	6	2017
Zhang Y	6	6	0.261	317	6	2004
Li H	5	9	0.333	452	9	2012
Krezel W	5	7	0.167	303	7	1997

NP = number of publication, PY_start = publication year, TC = total citation.

### 3.4. Institution analysis

The top 10 contributing institutions are presented in Table 3. Analysis of research output from institutions in the field of “Retinoic Acid Receptors and Neurodegenerative Diseases” reveals a decentralized knowledge production landscape characterized by international distribution and collaboration. In terms of institutional output distribution, the United States holds a dominant position (accounting for 6 of the top 10 institutions), while Europe (France, Sweden, and the United Kingdom) also contributes steadily. The institution with the highest publication volume (the University of California system) accounts for only 1.05% of total publications, indicating a low concentration index. This suggests relatively dispersed research capabilities and the absence of significant knowledge monopolization. Institutionally, university systems and national research organizations (such as NIH, Inserm, and CNRS) jointly constitute the primary research entities, reflecting cross-sectoral and multidisciplinary participation. Overall, the literature production structure in this field suggests it is in a growth and collaborative integration phase, demonstrating significant potential for transnational cooperation. Future research should further examine the evolution of its collaborative networks and the characteristics of knowledge flows.

**Table 3:** The top 10 contributing institutions.

Institutions	Articles	%	Country
University of California System	36	1.05%	America
Institut National De La Sante Et De La Recherche Medicale (Inserm)	34	0.99%	France
Karolinska Institutet	26	0.76%	Sweden
University of London	23	0.67%	England
Centre National De La Recherche Scientifique (Cnrs)	22	0.64%	France
Texas Tech University System	22	0.64%	America
Harvard University	21	0.61%	America
Johns Hopkins University	21	0.61%	America
National Institutes of Health, NIH	21	0.61%	America
Texas Tech University Health Sciences Center Lubbock	19	0.55%	America

**Figure 5.**
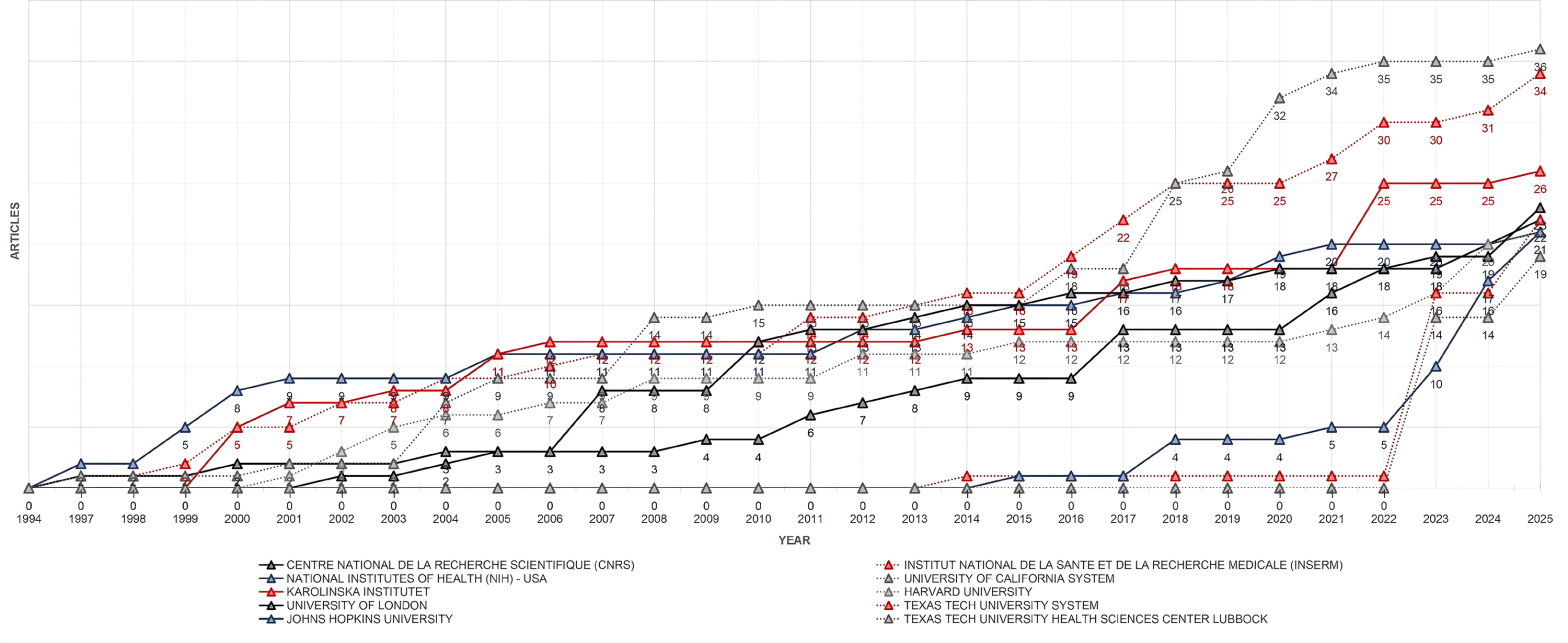
Over time, the top 10 institutions changed regarding the number of publications they published.

A bibliometric analysis of publication outputs from 1994 to 2025 reveals a substantial and sustained expansion in research focusing on RARs and NDs (Fig. 5). The research landscape is characterized by a marked expansion in scientific output over the past 3 decades, reflecting growing interest and investment in the role of RA signaling in neurodegenerative pathologies. The University of California System and the Institut National de la Santé et de la Recherche Médicale (INSERM) have emerged as the most prolific contributors, with sustained and substantial publication volumes by 2025 (36 and 34 articles, respectively). Notably, several institutions – including the Texas Tech University System and Johns Hopkins University – exhibited pronounced acceleration in publication activity in recent years, particularly after 2023, suggesting a shifting focus or intensified research efforts within these organizations. In contrast, historically active entities such as the National Institutes of Health (NIH) and the Centre National de la Recherche Scientifique (CNRS) demonstrated more gradual growth trajectories. The data reveal a dynamic and increasingly decentralized knowledge production network, with both traditional leaders and newly active institutions driving cumulative output. The overall trend indicates not only consistent growth in research volume but also a diversification of contributing centers, potentially reflecting broader interdisciplinary engagement and collaborative ventures in this translational research domain.

### 3.5. Keyword co-occurrence and analysis

**Figure 6.**
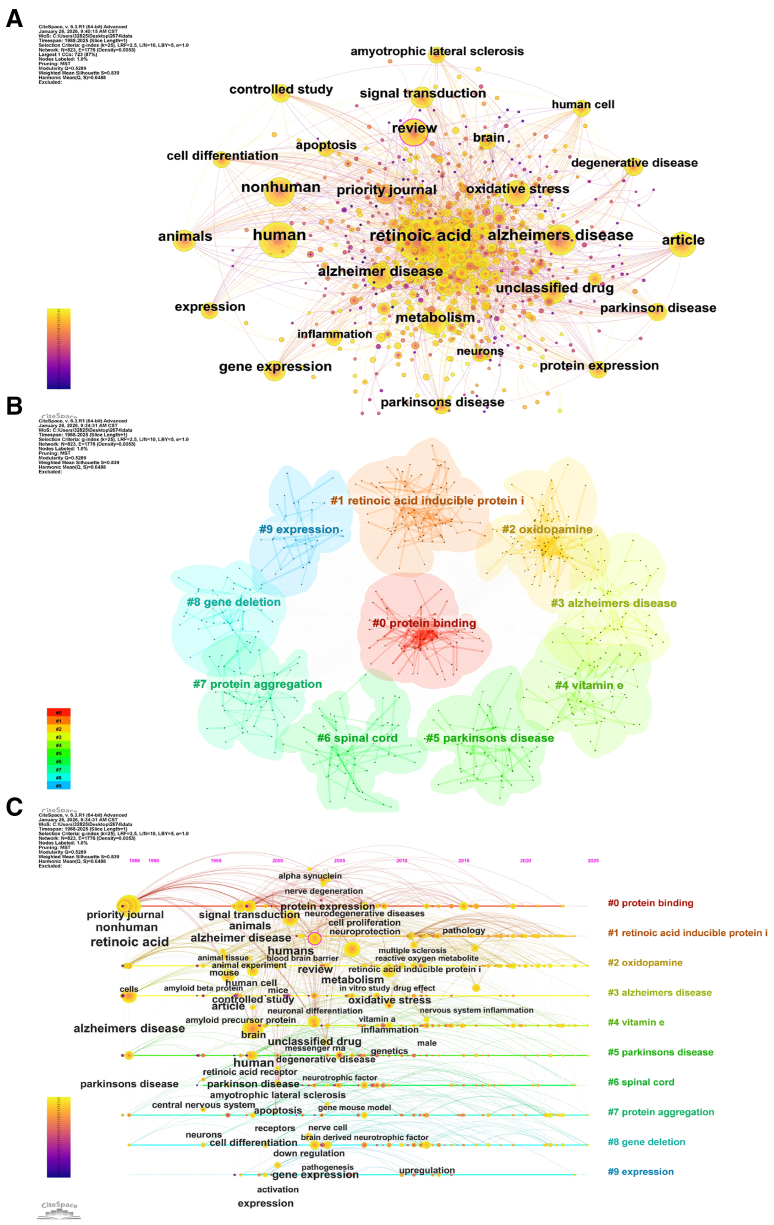
Cluster analysis networks and keyword co-occurrence maps. (A) Keyword co-occurrence network. Node size indicates keyword frequency, ring color (cool-to-warm) shows temporal activity, and connection thickness reflects co-occurrence strength. (B) Keyword clustering network. The network is partitioned into 10 colored clusters, representing thematic groups (e.g., #0: protein binding). The high modularity (*Q* = 0.53) indicates significant cluster structure. (C) Timeline view of keyword cluster evolution. Timeline of thematic clusters. The horizontal axis is time (1988–2025), and the vertical axis lists topic clusters. Each colored line represents a cluster, with dots (keywords) positioned at their year of first appearance in the literature.

Researchers can find key concepts and trends in a field using keyword co-occurrence analysis (Fig. 6A). Every node featuring vibrant yearly rings symbolizes a specific term. The nodes’ sizes correspond to the publications’ outputs. The distinct sections composed of nodes and links show the relationships between various keywords.A keyword co-occurrence network (N = 823, E = 1776, density = 0.0053) was constructed to map the intellectual landscape of RA research in NDs. The high modularity (*Q* = 0.5289) and silhouette value (*S* = 0.839) indicate a well-structured network with coherent thematic clusters. Retinoic acid emerges as the central hub with the highest betweenness centrality, bridging disease-specific research (Alzheimer, Parkinson, amyotrophic lateral sclerosis) and molecular mechanisms (gene expression, oxidative stress, inflammation). Thematic analysis reveals key clusters centered on proteostasis and Alzheimer pathology, cytoprotective signaling via pathways like nuclear factor erythroid 2-related factor 2/RAR, and neuroimmunology involving microglial activation. Temporal color mapping shows recent bursts (2020–2025) around nodes for amyotrophic lateral sclerosis and oxidative stress, indicating a shift from traditional Alzheimer-focused studies toward exploring RA’s role in broader neurodegenerative mechanisms and redox biology. This reflects an evolving paradigm from disease association to mechanism-driven intervention strategies centered on RA-mediated transcriptional regulation.

The keywords of the published articles were clustered using log-likelihood tests and CiteSpace software to investigate the study theme (Fig. 6B). Various colored nodes and edges correspond to distinct groupings. It visualizes the keyword co-occurrence network of the field (N = 823, E = 1776), derived from CiteSpace analysis of 2674 publications (1988–2025). The distinct color-coded clusters, validated by high modularity (*Q* = 0.5289) and silhouette (*S* = 0.839), represent core research themes. For instance, the “alzheimers disease” and “parkinsons disease” clusters map to major neurodegenerative research fronts, while “vitamin e” and “oxidopamine” link to oxidative stress mechanisms. Node proximity and link thickness indicate conceptual relatedness and co-occurrence strength. The strategic exclusion of generic terms (e.g., “protein binding”) clarifies domain-specific frontiers like “retinoic acid inducible protein i” and “protein aggregation.” Thus, the figure serves as a structural map, delineating the field’s intellectual architecture and thematic interconnections.

The keyword co-occurrence network visualized in Figure 6C was constructed using CiteSpace (v. 6.3.R1) based on data spanning 1988 to 2025 (N = 823, *E* = 1776, Density = 0.0053). It reveals a coherent yet dynamically evolving intellectual structure in the field, which is supported by strong clustering metrics (modularity *Q* = 0.5289; weighted mean silhouette *S* = 0.839). In this timeline visualization, the horizontal axis represents the time dimension (1988–2025), while the vertical axis lists the labels of individual thematic clusters. Distinct color codes correspond to each cluster, with every colored horizontal line mapping the evolution of a specific research theme. Nodes positioned along these timelines indicate the core keywords within each cluster, and their horizontal placement marks the year of the keyword’s first occurrence in the literature. Node size typically denotes frequency or betweenness centrality, serving to highlight pivotal concepts, while connections between nodes reflect co-occurrence strength, indicating established conceptual linkages. This view not only records the temporal shifts in research focus but also maps the deeper logic of disciplinary development. Taking the “Retinoic Acid Receptor (RAR)” related cluster as an illustrative example, its timeline reveals a paradigm shift from the exploration of basic molecular mechanisms toward an understanding of complex systemic diseases. Early nodes within this cluster are concentrated around terms such as “cells,” “Alzheimer disease,” and “Parkinson disease,” corresponding to the initial phase of research focused on RAR’s role in neurological contexts. Over time, the emergence of newer keywords like “cell proliferation neuroprotection,” “blood-brain barrier,” “oxidative stress,” and “genetics” signals an evolution in research emphasis – transitioning from developmental biology toward investigating its role in neuroplasticity and the pathophysiology of neurodevelopmental disorders. This trajectory visually encapsulates the field’s effort to bridge classical molecular signaling pathways with contemporary theories of neurodiversity. However, this visual progression also implies potential challenges. Despite the deepening investigation into RAR signaling mechanisms, if its conceptual links to application-oriented themes such as “drug discovery” or “translational medicine” appear relatively sparse or emerge only later in the timeline, it may expose a translational gap between intricate molecular mechanisms and heterogeneous clinical applications. This invites critical reflection: future research must extend beyond linear descriptions of isolated pathways, focusing more on their function within specific neural circuits, their interaction with other risk networks, and inter-individual genetic variation to genuinely connect fundamental discoveries with clinical practice. The network’s capacity to reflect both historical progression and thematic structure is underpinned by the analytical parameters employed in its construction, including the g-index (*k* = 25), the LRF (local citation frequency threshold), L/N (terms per slice), and pruning via the Minimum Spanning Tree algorithm. These parameters collectively ensure a structurally meaningful and historically sensitive extraction of research fronts, rendering the figure not merely a thematic index but an analytical map for interpreting the field’s internal logic and future trajectory.

**Figure 7.**
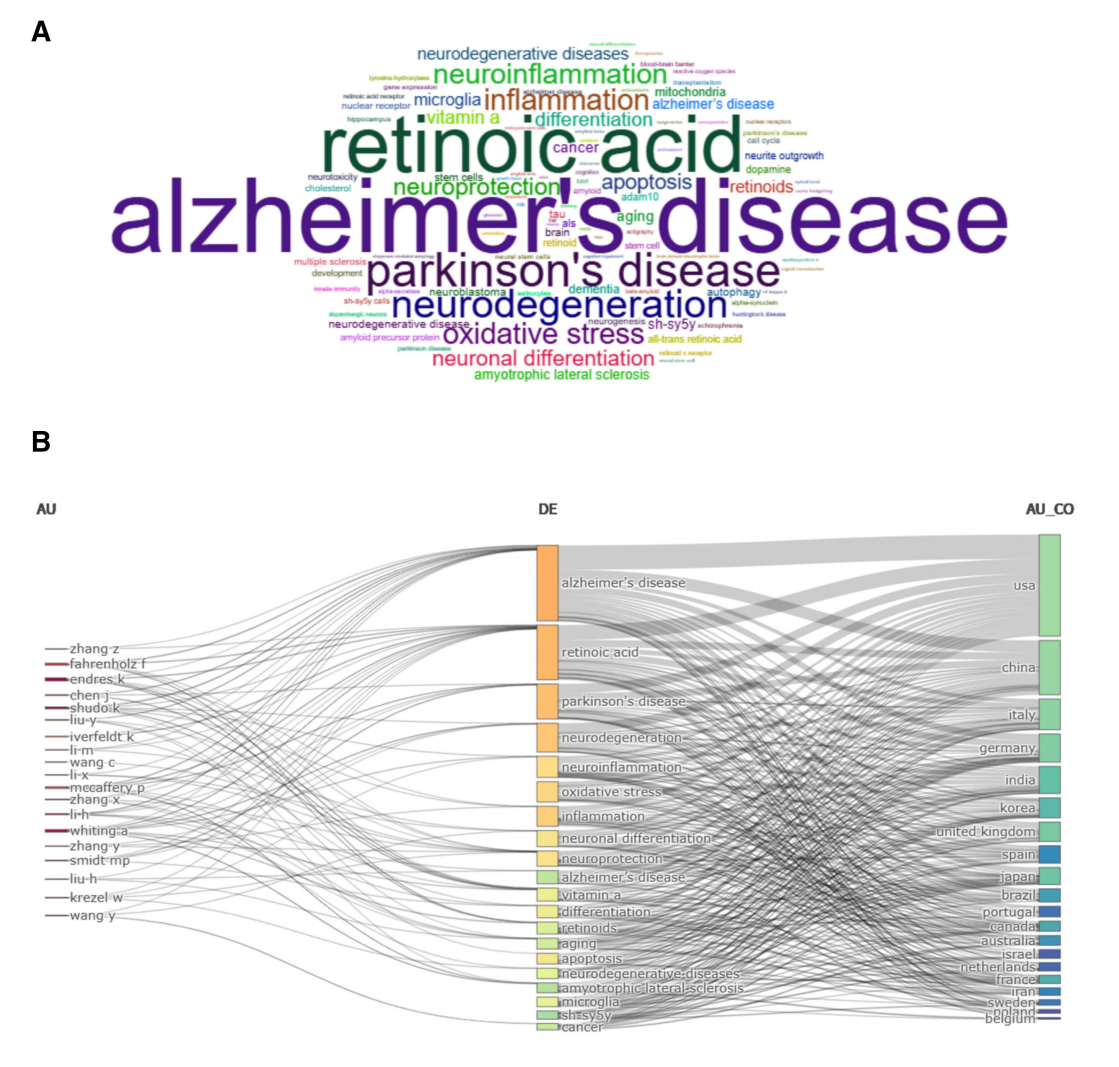
The author’s word cloud and 3-field plot about RARs in NDs. (A) Word cloud of high-frequency keywords. Word size represents frequency; color and position are for visual distinction and layout only. (B) Three-field plot of RAR research. Author (AU), Keywords (DE), Country/region (AU_CO); The lines represent the co-occurrence relationship of cross-dimensional entries, and the thicker the line, the stronger the association.

Utilizing the R package bibliometrix, an analysis of high-frequency keywords was conducted (Fig. 7A). Distinct colors correspond to distinct keywords, while keyword frequency is represented by size. The resulting word cloud, centered on “Alzheimer disease,” clearly maps the complex and dynamic research landscape in this field. The prominence of terms like “neuroinflammation” and “microglial inflammation” signals a paradigm shift: the research focus is moving from the traditional dominance of the “amyloid cascade hypothesis” towards a new stage centered on the neuroimmune system. The academic community increasingly recognizes that microglia are not passive responders but active regulators in disease progression. Their dysfunctional states are directly linked to synaptic loss and neuronal damage, making the targeting of microglial phenotypic switching a highly promising therapeutic strategy. Concurrently, “amyloid,” as a classic pathological hallmark, still holds a significant position. However, current research increasingly focuses on exploring its cascading and amplifying effects in conjunction with downstream events such as neuroinflammation, “oxidative stress,” and “apoptosis,” rather than viewing it in isolation. This integrated perspective is also reflected in the growing attention to neuroplasticity. The emergence of keywords like “neuronal differentiation” and “vitamin A” reveals an expanding research emphasis on early compensatory mechanisms and intrinsic repair potential in the disease process. Understanding how nutritional factors and intrinsic differentiation programs counteract degenerative progression has become an emerging hotspot. The appearance of “amyotrophic lateral sclerosis” and “cancer” in the word cloud profoundly reflects a trend towards interdisciplinary convergence. The former points to the exploration of common mechanisms, such as shared proteinopathies involving TAR DNA-binding protein 43. The latter stems from a biological interest in the seemingly opposing, yin-yang-like regulation of cell fate pathways (e.g., apoptosis/survival) in neurodegeneration versus cancer. These cross-disease comparisons aim to uncover more fundamental principles of cellular homeostasis. Furthermore, the presence of model system keywords like “SH-SY5Y” underscores the critical bridging role of experimental models in translating basic mechanistic insights towards clinical application. In summary, this word cloud vividly illustrates that research has entered an era of integration across multiple systems and scales. Future breakthroughs are likely to emerge at the intersection of neuroimmunology, metabolic regulation, proteostasis, and cellular regeneration. They will also depend on computational biology and multi-omics technologies to deeply deconstruct and quantitatively connect the complex knowledge network represented by these keywords.

The knowledge structure of the RAR research field was visualized using a 3-field plot (Fig. 7B). In the figure, the left, middle, and right fields correspond to authors (AU), keywords (DE), and author countries/regions (AU_CO). The thickness of the connecting lines represents co-occurrence strength, while color variation distinguishes different thematic clusters. The figure lists key themes including “Alzheimer disease,” “retinoic acid,” “Parkinson disease,” “neurodegeneration,” “neuroinflammation,” “oxidative stress,” and so on. It highlights a notable interdisciplinary trend in current neuroscience, particularly in neurodegeneration research: the potential role of RA and its related metabolites (such as vitamin A and retinoids) in neuroprotection, differentiation, and inflammation regulation has attracted broad attention. The frequent co-occurrence of these topics underscores the interactive relationships between nutritional factors, cellular stress mechanisms, and the pathology of neurological diseases, with a particular focus on neuroinflammation and oxidative stress as common pathways. The inclusion of terms such as “SH-SY5Y” and “microglia” further reflects a research continuum extending from in vitro models to neuroimmune mechanisms. Taken together, the network emphasizes a research framework that spans fundamental molecular mechanisms to systemic disease regulation, suggesting that future studies may move toward a multidimensional strategy integrating nutritional interventions, neuroimmune modulation, and validation through cellular models.

### 3.6. Theme evolution and thematic map

**Figure 8.**
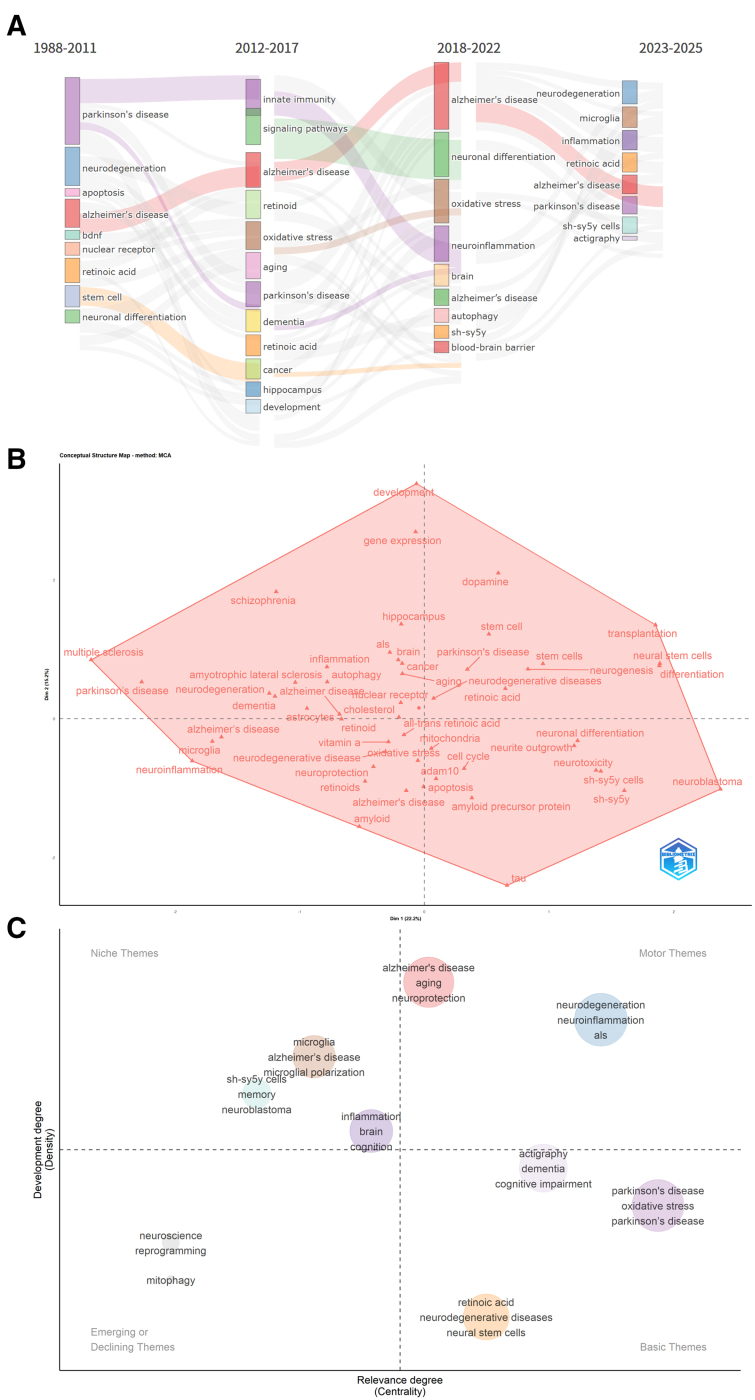
Thematic map and theme evolution. (A) Analysis of the evolution of keywords for RARs and NDs between 1988 and 2025. Timeline showing theme prominence, with point color indicating origin period and lines showing evolutionary links. (B) Map of conceptual structure. The clustering of all themes within the 2-dimensional space (37.4% variance explained) (C) Map with a theme. Themes are categorized into 4 quadrants (basic, motor, niche, emerging/declining).

Theme maps and theme evolution keywords can be used to reveal research hotspots, shifts in research interests, and key areas within a discipline. This reflects the developmental trends of the field and provides a basis for predicting future research directions. Thematic evolution analysis (Fig. 8A) delineates the shifting research landscape surrounding RARs within the neuroscience domain. The horizontal axis represents the chronological timeline (1988–2025), while the vertical axis denotes thematic intensity or frequency. Distinct color clusters correspond to major thematic foci, visually mapping the progression of research priorities. The early period (1988–2011) centered on neuronal differentiation and classical neurodegenerative mechanisms (e.g., BDNF, nuclear receptor). This transitioned to a cluster (2012–2017), highlighting neuroinflammation and oxidative stress pathways (e.g., innate immunity, hippocampus). The most recent phase (2018–2025) focuses on microglia, the blood–brain barrier, and translational disease models (e.g., SH-SY5Y cells, actigraphy), reflecting a paradigm shift toward integrated neuropathological systems. This evolution underscores how RAR research has expanded from singular receptor characterization to intersection with modern frameworks of neuroinflammation, autophagy, and the neurovascular unit. While this integration supports its therapeutic relevance in neurodiverse disorders, continued reliance on established cellular models (e.g., SH-SY5Y) may constrain understanding of cellular specificity and in vivo complexity. Future studies should incorporate spatial transcriptomics and clinical correlation to advance a more nuanced, systems-level perspective.

The conceptual structure map (Dim 1 explaining 22.2% and Dim 2 explaining 15.2% of the variance) revealed that keywords representing different thematic areas are tightly clustered in the coordinate space (all at 0.8, 0.8) (Fig. 8B). This forms a highly interconnected visual cluster, indicating that the RAR research field has not yet clearly differentiated into distinct subdomains but instead constitutes a strongly integrated knowledge network. In such maps, colors typically distinguish thematic clusters; their pronounced overlap here further underscores the field’s conceptual interdisciplinarity. Major clusters can be summarized as: neural development and stem cells (e.g., neurogenesis, differentiation), NDs (e.g., Alzheimer, Parkinson), cellular and molecular mechanisms (e.g., autophagy, neuroinflammation), and RAR-specific signaling and models (e.g., retinoid, SH-SY5Y cells). The integration observed across these thematic clusters is supported by fundamental RAR signaling pathways that function as common mechanistic links. For instance, the close association between ND and molecular mechanisms is clearly demonstrated by the role of RARα in transcriptionally upregulating ADAM10. As a key α-secretase, ADAM10 shifts amyloid precursor protein processing toward the nonamyloidogenic pathway, thereby lowering β-amyloid production in models of Alzheimer disease.^[18,19]^ Similarly, the overlapping of developmental, degenerative, and cellular-mechanism themes reflects the context-dependent functions of processes such as autophagy and neuroinflammation, both finely tuned by RAR signaling. During neural development, these processes aid in neuronal differentiation and synaptic pruning, whereas in neurodegeneration they contribute to clearing pathogenic protein aggregates and mitigating chronic inflammation.^[20,21]^ Thus, the strong thematic cohesion seen in the map corresponds to the central, multifunctional role of RAR signaling across different physiological and pathological states. This structure reflects the translational character of RAR research, with mechanisms spanning neurodevelopment, degeneration, and repair, highlighting its potential as a therapeutic target for neural diversity. However, the high co-occurrence of themes may also suggest that current research remains conceptually dense yet structurally undifferentiated. Future studies employing temporal analysis and finer subcluster resolution are needed to better elucidate the specific pathways of RAR signaling in neuropathophysiology and its prospects for clinical translation.

A thematic map was used to evaluate certain keywords’ significance and potential future trends (Fig. 8C). The map utilizes centrality (*x*-axis) and density (*y*-axis) as analytical dimensions, which respectively reflect a theme’s importance within the scientific network and its internal coherence or developmental maturity. Different thematic categories – such as motor themes, emerging/declining themes, and basic themes – are typically distinguished by color, while node size generally corresponds to publication volume or influence. The analysis reveals that all identified themes exhibit centrality and density values close to 1.0, indicating that they are positioned within high-visibility, well-developed research clusters. These themes encompass major neurodegenerative disorders (e.g., Alzheimer and Parkinson diseases), core pathological mechanisms (neuroinflammation, oxidative stress, mitophagy), and widely adopted cellular models such as SH-SY5Y cells. Notably, the RA theme – associated with neuroprotection and cellular reprogramming – also resides within this central cluster, highlighting its integrative potential within disease regulatory networks. Mechanistically, this potential is rooted in the pathway’s dual role in modulating key pathological processes. Regarding oxidative stress, RA signaling mitigates neuronal oxidative damage by activating the nuclear factor erythroid 2-related factor 2/antioxidant response element antioxidant pathway, suppressing the NF-κB inflammatory pathway, and upregulating endogenous antioxidant enzymes such as superoxide dismutase and catalase.^[22–24]^ Retinoic acid itself, primarily through its receptor heterodimer RARα/RXR, regulates the transcription of downstream target genes (e.g., BDNF, GDNF), thereby promoting neuronal survival and synaptic plasticity while inhibiting key pathological processes including β-amyloid accumulation and tau hyperphosphorylation.^[13,25,26]^ In terms of neuroinflammation, RARs modulate the activation states of microglia and astrocytes, inhibit the assembly of the NLRP3 inflammasome, and consequently reduce the release of pro-inflammatory cytokines such as interleukin-1 beta and tumor necrosis factor-alpha.^[27–29]^ From a critical perspective, the convergence of theme metrics may indicate a paradigm focused on established mechanisms and models, reflecting a degree of intellectual path dependency. Although RA signaling has been incorporated into the core research landscape, its mechanistic interactions with neuroimmune modulation and neurodiversity-informed therapeutic strategies remain insufficiently explored. Future research should prioritize the systematic integration of this pathway with dominant themes such as neuroinflammation, glial regulation, and cognitive preservation, thereby fostering a more interconnected and multi-target framework for therapeutic development in NDs.

## 4. Discussion

Through bibliometric research, we have depicted the academic structure and evolution of research on RARs in the context of NDs. The observed growth in scientific output and the formation of distinct yet interconnected international collaborative networks reflect a maturing, interdisciplinary field transitioning from foundational molecular biology toward integrative pathophysiological inquiry.

The sustained leadership of the United States in both productivity and citation influence is likely attributable to its concentrated funding ecosystems, established research infrastructure, and the presence of large, collaborative academic networks. The notable rise of China in publication volume aligns with its increased strategic investment in neuroscience, though its lower average citation rate may indicate a focus on rapid output or research themes still gaining global traction, compared to the more established, high-impact work from nations like France and Germany.

Keyword and thematic evolution reveal a critical shift in research priorities. The early dominance of terms like “neuronal differentiation” and “gene expression” has progressively given way to clusters centered on “neuroinflammation,” “oxidative stress,” “microglia,” and the “blood–brain barrier.” This trajectory mirrors the broader paradigm shift in neuroscience away from a purely neuron-centric view and towards recognizing the essential roles of neuroimmune crosstalk, glial dysfunction, and systemic factors in neurodegeneration. RAR research has effectively converged with these central themes, positioning retinoid signaling not merely as a developmental pathway but as a modulator of the inflammatory and oxidative landscapes that drive disease progression.

The linkage between RAR mechanisms and the emerging focus on neurodiversity therapeutics represents a significant, though still developing, frontier. The co-occurrence analysis shows strong conceptual proximity between neurodevelopmental processes (e.g., neurogenesis, synaptic plasticity) and psychiatric/neurodegenerative disorders. This suggests that RARs, as key regulators of neural development and plasticity, may sit at a critical nexus. Their dysfunction could contribute to a “developmental vulnerability” that manifests later as neurodegeneration or neuropsychiatric illness. However, the relative peripherality of explicitly translational keywords indicates that this mechanistic understanding has not yet been fully operationalized into targeted therapeutic strategies for neurodiverse conditions. The field currently excels at modeling RAR roles in disease pathology but appears less focused on leveraging this knowledge for intervention in disorders of neural development and function.

A critical assessment of the collaboration networks reveals a robust but modularized global effort. While the high interconnectedness of major hubs like the USA fosters knowledge diffusion, the clear community structure suggests that collaboration often occurs within established geographic or thematic circles. Enhancing inter-modular links could accelerate the cross-fertilization of ideas, for instance, by more deeply integrating European strengths in molecular pharmacology with North American capacities in translational disease modeling and Asian research scales.

Researchers looking into this area can use the current study as a reference because it offers the first thorough, impartial, and logical examination of the literature on RARs and NDs and their development trends. Furthermore, we looked into the many dimensions of research hotspots using various bibliometric software programs to get more precise and unbiased results.

## 5. Limitations

This study has several limitations. First, the literature search was confined to 3 major databases—WoS Core Collection, Scopus, and PubMed—and did not include other sources such as Dimensions. As a result, some studies not indexed in these selected databases may have been missed. Nevertheless, the combined use of these 3 databases has provided a sufficiently comprehensive and representative dataset for analyzing research trends in RARs and NDs. Second, the bibliometric analysis relied primarily on traditional indicators such as total citation counts, which are strongly influenced by the publication date of the articles. Future studies could employ time-normalized metrics – such as the average annual citation rate, relative citation ratio, or field-normalized citation impact – to allow a more nuanced and equitable comparison of the sustained influence of research outputs from different periods. Furthermore, although basic data cleaning and author name normalization were performed, challenges remain in merging records across databases and fully disambiguating author identities – for example, in handling abbreviated names, name variants, or researchers with similar names. These issues may introduce minor inaccuracies in the productivity statistics of individual authors. Finally, bibliometric methods themselves cannot distinguish between studies focused on human versus animal models, nor can they directly assess the scientific quality or translational potential of the research. Despite these constraints, the present analysis still offers an objective overview of the overall development and knowledge structure of the field. Researchers in related areas may use this study to identify current hotspots and evolving trajectories, thereby fostering deeper investigation into the role of RARs in NDs.

## 6. Conclusion

In summary, RAR research in neurodegeneration is a vibrant and evolving field, successfully integrating itself into contemporary neuroscience frameworks centered on neuroinflammation and glial biology. The United States remains the central contributor, but the landscape is becoming increasingly globalized and collaborative. The intellectual evolution from receptor-centric studies to systems-level pathophysiology underscores the growing recognition of RARs’ pleiotropic roles in brain health and disease.

The most promising, yet still underexplored, direction lies in bridging the field’s deep mechanistic knowledge with the therapeutic challenges of neurodiversity. RAR pathways, governing both developmental patterning and homeostatic responses in the adult brain, offer a unique conceptual link between neurodevelopmental origins and neurodegenerative endpoints. Future progress will depend on deliberately extending inquiry beyond classical neurodegeneration models to include neurodevelopmental disorder paradigms, while employing advanced tools like single-cell omics and humanized model systems. To realize the translational potential, the field must move beyond describing RARs’ involvement in pathology and toward designing precise pharmacological or genetic strategies to modulate this signaling axis for neuroprotection, repair, and cognitive resilience across the lifespan.

## Author contributions

**Conceptualization:** Wei Cui, Xiang Wu.

**Data curation:** Ting Wu, Tengyu Zhang.

**Formal analysis:** Ting Wu.

**Funding acquisition:** Xiaojie Zhai, Xiang Wu.

**Investigation:** Ahmad Khaled Harb.

**Methodology:** Xiaojie Zhai, Yunfei Cao.

**Project administration:** Wei Cui, Xiang Wu.

**Resources:** Wei Cui.

**Software:** Ting Wu, Tengyu Zhang, Yunfei Cao.

**Supervision:** Ahmad Khaled Harb, Wei Cui, Yunfei Cao.

**Validation:** Tengyu Zhang.

**Visualization:** Ting Wu.

**Writing – original draft:** Ting Wu.

**Writing – review & editing:** Ahmad Khaled Harb, Xiang Wu.





## References

[1] Agnello L Ciaccio M. Neurodegenerative diseases: from molecular basis to therapy. Int J Mol Sci. 2022;23:12854. 36361643 10.3390/ijms232112854 PMC9654859

[2] Alrouji M Anwar S Venkatesan K. Iron homeostasis and neurodegeneration in the ageing brain: Insight into ferroptosis pathways. Ageing Res Rev. 2024;102:102575. 39515619 10.1016/j.arr.2024.102575

[3] Young AP Denovan-Wright EM. Microglia-mediated neuron death requires TNF and is exacerbated by mutant Huntingtin. Pharmacol Res. 2024;209:107443. 39362509 10.1016/j.phrs.2024.107443

[4] Van Schependom J D’Haeseleer M. Advances in neurodegenerative diseases. J Clin Med. 2023;12:1709. 36902495 10.3390/jcm12051709 PMC10002914

[5] Tambones I Sagar A Vankova P. New structural insights into the control of the retinoic acid receptors RAR/RXR by DNA, ligands, and transcriptional coregulators. Nucleic Acids Res. 2025;53:gkaf967. 41036627 10.1093/nar/gkaf967 PMC12489474

[6] Moreno-Oñate M Gallardo-Fuentes L Martínez-García PM. Rewiring of the epigenome and chromatin architecture by exogenously induced retinoic acid signaling during zebrafish embryonic development. Nucleic Acids Res. 2024;52:3682–701. 38321954 10.1093/nar/gkae065 PMC11040003

[7] Pignolo RJ Pacifici M. Retinoid agonists in the targeting of heterotopic ossification. Cells. 2021;10:3245. 34831466 10.3390/cells10113245 PMC8617746

[8] Larange A Takazawa I Kakugawa K. A regulatory circuit controlled by extranuclear and nuclear retinoic acid receptor α determines T cell activation and function. Immunity. 2023;56:2054–69.e10. 37597518 10.1016/j.immuni.2023.07.017 PMC10552917

[9] Saha P Talwar P. Identification of PPREs and PPRE associated genes in the human genome: insights into related kinases and disease implications. Front Immunol. 2024;15:1457648. 39434882 10.3389/fimmu.2024.1457648 PMC11491715

[10] Tian Y Liu B Li Y. Activation of RARα receptor attenuates neuroinflammation after SAH via promoting M1-to-M2 phenotypic polarization of microglia and regulating Mafb/Msr1/PI3K-Akt/NF-κB pathway. Front Immunol. 2022;13:839796. 35237277 10.3389/fimmu.2022.839796 PMC8882645

[11] Karova K Polcanova Z Knight L. Hyperactive delta isoform of PI3 kinase enables long-distance regeneration of adult rat corticospinal tract. Mol Ther. 2025;33:752–70. 39748509 10.1016/j.ymthe.2024.12.040 PMC11852985

[12] Xie X Li C Yu J. MTHFD1 is critical for the negative regulation of retinoic acid receptor signalling in anencephaly. Brain. 2023;146:3455–69. 36928982 10.1093/brain/awad084

[13] Almaguer J Hindle A Lawrence JJ. The contribution of hippocampal all-trans retinoic acid (ATRA) deficiency to Alzheimer’s disease: a narrative overview of ATRA-dependent gene expression in post-mortem hippocampal tissue. Antioxidants (Basel). 2023;12:1921. 38001775 10.3390/antiox12111921 PMC10669734

[14] Ciancia M Rataj-Baniowska M Zinter N. Retinoic acid receptor beta protects striatopallidal medium spiny neurons from mitochondrial dysfunction and neurodegeneration. Prog Neurobiol. 2022;212:102246. 35151792 10.1016/j.pneurobio.2022.102246

[15] Chen Y Zhou Y Cai J. The activation of RARα prevents surgery-induced cognitive impairments via the inhibition of neuroinflammation and the restoration of synaptic proteins in elderly mice. Int Immunopharmacol. 2024;130:111772. 38432148 10.1016/j.intimp.2024.111772

[16] Li X Xu D Ding C. Comparative analysis of domestic and foreign coal mine safety supervision modes based on knowledge map. Environ Sci Pollut Res Int. 2023;30:89535–47. 37453014 10.1007/s11356-023-27976-1

[17] Beckman TJ. Evaluating academic physicians’ bibliographies: criteria and fairness. Am J Med. 2025;138:34–8. 39147087 10.1016/j.amjmed.2024.08.015

[18] Musardo S Therin S Pelucchi S. The development of ADAM10 endocytosis inhibitors for the treatment of Alzheimer’s disease. Mol Ther. 2022;30:2474–90. 35390543 10.1016/j.ymthe.2022.03.024 PMC9263258

[19] Nakamura M Li Y Choi BR. GDE2-RECK controls ADAM10 α-secretase-mediated cleavage of amyloid precursor protein. Sci Transl Med. 2021;13:585. 10.1126/scitranslmed.abe6178 PMC8085789 33731436

[20] Wan L Jia RM Ji LL. AMPK-autophagy-mediated inhibition of microRNA-30a-5p alleviates morphine tolerance via SOCS3-dependent neuroinflammation suppression. J Neuroinflammation. 2022;19:25. 35093117 10.1186/s12974-022-02384-3 PMC8800317

[21] Wang L Tian S Ruan S. Neuroprotective effects of cordycepin on MPTP-induced Parkinson’s disease mice via suppressing PI3K/AKT/mTOR and MAPK-mediated neuroinflammation. Free Radic Biol Med. 2024;216:60–77. 38479634 10.1016/j.freeradbiomed.2024.02.023

[22] Wang Q Botchway BOA Zhang Y Liu X. Ellagic acid activates the Keap1-Nrf2-ARE signaling pathway in improving Parkinson’s disease: a review. Biomed Pharmacother. 2022;156:113848. 36242848 10.1016/j.biopha.2022.113848

[23] Lai X Wu A Bing Y. Retinoic acid protects against lipopolysaccharide-induced ferroptotic liver injury and iron disorders by regulating Nrf2/HO-1 and RARβ signaling. Free Radic Biol Med. 2023;205:202–13. 37302616 10.1016/j.freeradbiomed.2023.06.003

[24] Amoroso R Maccallini C Bellezza I. Activators of Nrf2 to counteract neurodegenerative diseases. Antioxidants (Basel). 2023;12:778. 36979026 10.3390/antiox12030778 PMC10045503

[25] Shibata M Pattabiraman K Lorente-Galdos B. Regulation of prefrontal patterning and connectivity by retinoic acid. Nature. 2021;598:483–8. 34599305 10.1038/s41586-021-03953-x PMC9018119

[26] Duester G. Insufficient support for retinoic acid receptor control of synaptic plasticity through a non-genomic mechanism. Front Neuroendocrinol. 2023;71:101099. 37647946 10.1016/j.yfrne.2023.101099 PMC10840951

[27] Ganz T Fainstein N Elad A. Microbial pathogens induce neurodegeneration in Alzheimer’s disease mice: protection by microglial regulation. J Neuroinflammation. 2022;19:5. 34991645 10.1186/s12974-021-02369-8 PMC8740456

[28] Chen X Wang Y Huang J. Tamibarotene directly targets the NACHT domain of NLRP3 to alleviate acute myocardial infarction. Biochem Pharmacol. 2025;234:116801. 39952330 10.1016/j.bcp.2025.116801

[29] Santos-García I Bascuñana P Brackhan M. The ABC transporter A7 modulates neuroinflammation via NLRP3 inflammasome in Alzheimer’s disease mice. Alzheimers Res Ther. 2025;17:30. 39871385 10.1186/s13195-025-01673-2 PMC11773842

