# Supplementary material for: Research on RARs in neurodegenerative diseases: A bibliometric analysis
Source: Medicine (Baltimore). 2026 Jun 26;105(26):e49472. doi: 10.1097/MD.0000000000049472 (PMC13313660; doi:10.1097/MD.0000000000049472)
Supplement: Supplementary file 1 [file medi-105-e49472-s001.docx]

**Supplementary File 1. Search expressions for each database**

**WOS:**

TS=((retinoic acid receptor*) OR (retinoic acid-binding protein*) OR (retinoic acid signaling) OR RAR OR RARS) AND TS=((neurodegenerative disease*) OR (neurodegenerative disorder*) OR alzheimer* OR parkinson* OR huntington* OR "amyotrophic lateral sclerosis" OR ALS OR "Lou Gehrig's disease")

**SCOPUS:**

TITLE-ABS-KEY((retinoic acid receptor*) OR (retinoic acid-binding protein*) OR (retinoic acid signaling) OR RAR OR RARS) AND TITLE-ABS-KEY((neurodegenerative disease*) OR (neurodegenerative disorder*) OR alzheimer* OR parkinson* OR huntington* OR "amyotrophic lateral sclerosis" OR ALS OR "Lou Gehrig's disease")

**PUBMED:**

(("retinoic acid receptor*"[Title/Abstract] OR "retinoic acid-binding protein*"[Title/Abstract] OR "retinoic acid signaling"[Title/Abstract] OR RAR[Title/Abstract] OR RARS[Title/Abstract]) AND ("neurodegenerative disease*"[Title/Abstract] OR "neurodegenerative disorder*"[Title/Abstract] OR alzheimer*[Title/Abstract] OR parkinson*[Title/Abstract] OR huntington*[Title/Abstract] OR "amyotrophic lateral sclerosis"[Title/Abstract] OR ALS[Title/Abstract] OR "Lou Gehrig's disease"[Title/Abstract]))

**search condition：**

English / article and review /Without time limit

**Initial quantity：**

Total amount of literature：1663

Number of WOSCC: 693

Number of SCOPUS: 798

Number of PUBMED: 172

**The number of documents after database integration:**

After merging and removing duplicates (based on WOS, first removing duplicate items from Scopus, and then using the merged and duplicate-free results from WOS and Scopus as the standard to remove PM):

Total amount of literature: 1094

Number of WOS: 676

Number of SCOPUS: 410

Number of PM: 8

To obtain comprehensive and reliable literature data, this study simultaneously utilized three databases—Web of Science Core Collection (WOSCC), Scopus, and PubMed—as primary search sources. WOSCC is widely used in medical and life sciences research due to its high-quality, structured citation system. This study exclusively incorporates the Science Citation Index Expanded (SCIE) and Emerging Sources Citation Index (ESCI) to ensure the academic representativeness and consistency of the included literature. As one of the world's most comprehensive peer-reviewed literature databases, Scopus provides interdisciplinary and systematic literature support, thereby expanding the scope of this study's search. PubMed, developed by the U.S. National Library of Medicine (NLM), is the most central literature database in the biomedical field and a vital source for highly relevant medical literature. Therefore, conducting a systematic search across these three databases helps comprehensively present the full landscape of research developments in tumor-associated bacteria and tumor immunotherapy.

Based on the selected databases, this study constructed independent yet structurally consistent retrieval strategies across the three databases. The retrieval themes centered on two core concepts—“retinoic acid receptors” and “neurodegenerative diseases”—with term combinations built using wildcards and Boolean logic to maximize coverage of relevant term variants. To enhance topic relevance, the search scope was uniformly restricted to the title, abstract, and author keywords fields. Additionally, automatically generated index terms (e.g., WOSCC's Keywords Plus) were excluded to prevent bias introduced by non-manually annotated terms. The WOSCC search was limited to the TS field; Scopus covered the TITLE-ABS-KEY fields; PubMed was restricted to the Title/Abstract fields. All searches were restricted to English-language literature and included only Article and Review types. The final search date was January 21, 2026. Complete search terms are detailed in Supplementary Material Table S1.

This search yielded 1,663 initial documents (WOSCC: 693; Scopus: 798; PubMed: 172). Two researchers (A and B) then independently screened all documents for relevance. Any disagreements during screening were resolved by a third researcher (C), who made the final decision. After excluding irrelevant studies, the literature data were downloaded in plain text and CSV formats, including comprehensive information such as titles, authors, institutions, countries, publication years, abstracts, keywords, and references. To standardize data structure, Python (version 3.11) was used to convert Scopus CSV files into plain text formats consistent with WOSCC and PubMed (full-record and reference formats). Data cleaning was also performed using Python (version 3.11), involving the following key steps: Removing records with “[Anonymous]” in the author field; Excluding virtual institutions such as “Egyptian Knowledge Bank (EKB)”; and Merging duplicate institutional names. Ultimately, 1,094 documents (WOSCC: 676; Scopus: 410; PubMed: 8) were included for subsequent bibliometric analysis.
